# Genetic Diversity of and Differentiation among Five Populations of Blunt Snout Bream (*Megalobrama amblycephala*) Revealed by SRAP Markers: Implications for Conservation and Management

**DOI:** 10.1371/journal.pone.0108967

**Published:** 2014-09-29

**Authors:** Wei Ji, Gui-Rong Zhang, Wei Ran, Jonathan P. A. Gardner, Kai-Jian Wei, Wei-Min Wang, Gui-Wei Zou

**Affiliations:** 1 Key Laboratory of Freshwater Animal Breeding, Ministry of Agriculture, College of Fisheries, Huazhong Agricultural University, Wuhan, P. R. China; 2 Freshwater Aquaculture Collaborative Innovation Centre of Hubei Province, Wuhan, P. R. China; 3 School of Biological Sciences, Victoria University of Wellington, Wellington, New Zealand; 4 Key Laboratory of Freshwater Biodiversity Conservation, Ministry of Agriculture, Yangtze River Fisheries Research Institute, Chinese Academy of Fishery Sciences, Wuhan, P. R. China; The Ohio State University, United States of America

## Abstract

The blunt snout bream (*Megalobrama amblycephala*) is an important freshwater aquaculture fish throughout China. Because of widespread introductions of this species to many regions, the genetic diversity of wild and natural populations is now threatened. In the present study, SRAP (sequence-related amplified polymorphism) markers were used to assess genetic diversity of blunt snout bream. Three natural populations (Liangzi Lake, Poyang Lake and Yuni Lake, one cultured population (Nanxian) and one genetic strain (‘Pujiang No. 1’) of blunt snout bream were screened with 88 SRAP primer combinations, of which 13 primer pairs produced stable and reproducible amplification patterns. In total, 172 bands were produced, of which 132 bands were polymorphic. Nei's gene diversity (*h*) and Shannon's information index (*I*) values provided evidence of differences in genetic diversity among the five populations (Poyang Lake>Liangzi Lake>Nanxian>‘Pujiang No. 1’>Yuni Lake). Based on cluster analysis conducted on genetic distance values, the five blunt snout bream populations were divided into three groups, Poyang Lake and Liangzi Lake (natural populations), Nanxian and ‘Pujiang No. 1’ (cultured population and genetically selected strain), and Yuni Lake (natural population). Significant genetic differentiation was found among the five populations using analysis of molecular variance (AMOVA), with more genetic divergence existing among populations (55.49%), than within populations (44.51%). This molecular marker technique is a simple and efficient method to quantify genetic diversity within and among fish populations, and is employed here to help manage and conserve germplasm variability of blunt snout bream and to support the ongoing selective breeding programme for this fish.

## Introduction

Freshwater fishes are very diverse given the relatively small global size of the freshwater habitat, but are among the most endangered organisms globally [1]. Many species are now considered to be under threat in terms of reductions in their natural distributional ranges, and also reductions in numbers and sizes of populations as a consequence of factors such as pollution, replacement by invasive or deliberately introduced species, over-fishing, the aquarium pet trade, river flow modification and habitat change. A large body of literature points to the effects of such threats in terms of both localised and global extinctions of many freshwater fish species on all human-inhabited continents [1]–[7]. It has been noted that freshwater habitats in general (i.e., as a broad ecosystem type) are particularly susceptible to climate change impacts, both direct and indirect, and that consideration of even the most optimistic climate change scenarios points to the likelihood of *ex-situ* management of many species for their survival [8]. At a lesser scale of threat, the deliberate human-mediated movement of fishes between previously isolated lakes or rivers as a fisheries management activity contributes to the eroding of genetic differences between intra-specific populations, and thereby threatens the genetic integrity and genetic adaptation of local fish populations [9], [10]. As a consequence, there is now an increasing awareness of the need to balance the protection and genetic integrity of freshwater fish populations against the need to exploit many such populations as an important source of protein for human populations [11].

The magnitude of the threat faced by freshwater fishes of the world is highlighted by two review papers [1], [3]. Both papers point out that freshwater fishes and their associated fisheries are important sources of employment, recreation and culture, and in developing regions they provide a significant contribution to the provision of local human food sources. Both papers also make the point that freshwater systems, because of their landscape positions, are subject to a number of externalities (threats not directly associated with or a function of the water body itself) that exacerbate problems of local fish endemism and non-substitutability. Cooke *et al.* note that many different groups and organizations have a role to play in conserving freshwater fishes and conclude by noting that failure to engage with the public will hinder conservation efforts and outcomes [1], while Dudgeon *et al.* had previously noted that threats to freshwater systems may constitute the greatest conservation challenge yet faced, and may require a new paradigm for biodiversity protection [3].

The Yangtze River is the world's third longest river system, with more than 3000 tributaries and 4000 lakes [2]. The system is notable for its speciose fish fauna and for high levels of endemism [2], [12]. However, it is also notable for the modifications that it has undergone and for the threats now faced by its biota ([2], [12] and references therein). The blunt snout bream (*Megalobrama amblycephala* Yih, 1955) (Teleostei: Cyprinidae) is an important endemic freshwater fish that has been widely favoured and is now cultured in China as a delicacy. It was originally distributed in the middle Yangtze River and a few accessory lakes, of which Yuni Lake, Liangzi Lake and Poyang Lake are the major sources [13]. Since having been recognized as a new species in the 1950s [14], this fish has become a principal species for freshwater aquaculture in China. This bream has been widely introduced all over the country because of its ease of culture, rapid growth rate, resistance to disease, high catchability and many other advantages [15]. In 2012, the total production of the bream reached 705,821 tonnes [16]. After domestication over a fifty year period, the aquaculture performance of many cultured populations has deteriorated as indicated by growth depression and the early onset of maturation. This deterioration is thought to be due to inbreeding and poor management of broodstocks [17]. Furthermore, as a consequence of environmental change and over-exploitation of resources, natural populations of this species have decreased substantially over the last few decades. As a consequence, the germplasm resources and gene pool diversity of this species in natural inland waters are now threatened by introductions of fish with unknown genetic histories, artificial propagation and poor stocking practices [13]. At the present time, because of these ongoing threats to the integrity of blunt snout bream populations, it is urgently required to clarify genetic diversity and population structure of this bream in order to effectively protect and utilize natural populations and their genetic resources.

Genetic diversity has been estimated in several natural and genetically selected populations of blunt snout bream using morphometrics and isozymes [13], RAPDs [18]–[20], mtDNA sequencing and RFLPs [21]–[23], and microsatellite markers [24]–[26]. For the first time however, we assess genetic diversity in natural populations of blunt snout bream and contrast this with genetic diversity estimates derived from a genetically selected strain and a cultured population. Sequence-related amplified polymorphism (SRAP) is a molecular marker system developed for selective amplification of open reading frames [27]. These polymorphisms result mainly from various promoters, introns and spacers among different species and individuals. SRAP is a highly reproducible and highly informative technique for assessing genetic diversity in comparison with other PCR-based techniques [28], [29]. SRAP markers have been successfully applied to the assessment of genetic diversity, strain identification and linkage map construction in a number of species, principally plants of commercial value [30]–[34]. More recently, SRAPs have also been applied to several aquatic animals [35]–[39] and have been shown to be highly informative and reliable markers for the evaluation of genetic diversity and population differentiation.

In this study, SRAP markers are used to investigate population genetic diversity and differentiation among three natural populations, a genetically selected strain and a cultured population of blunt snout bream in China. Our aim is to better understand the genetic diversity and integrity of natural populations based upon geography and the genetic affinities of selected strain and cultured populations based upon history. This work contributes to the critical need for germplasm conservation while at the same time supporting the ongoing selective breeding and improvement of blunt snout bream.

## Materials and Methods

### Ethics Statement

No specific permits were required for the field studies described here. The sampling lakes and ponds are not privately-owned or protected in any way, and the field sampling activities did not involve endangered or protected species.

### Sample collection and DNA extraction

**Figure 1:**
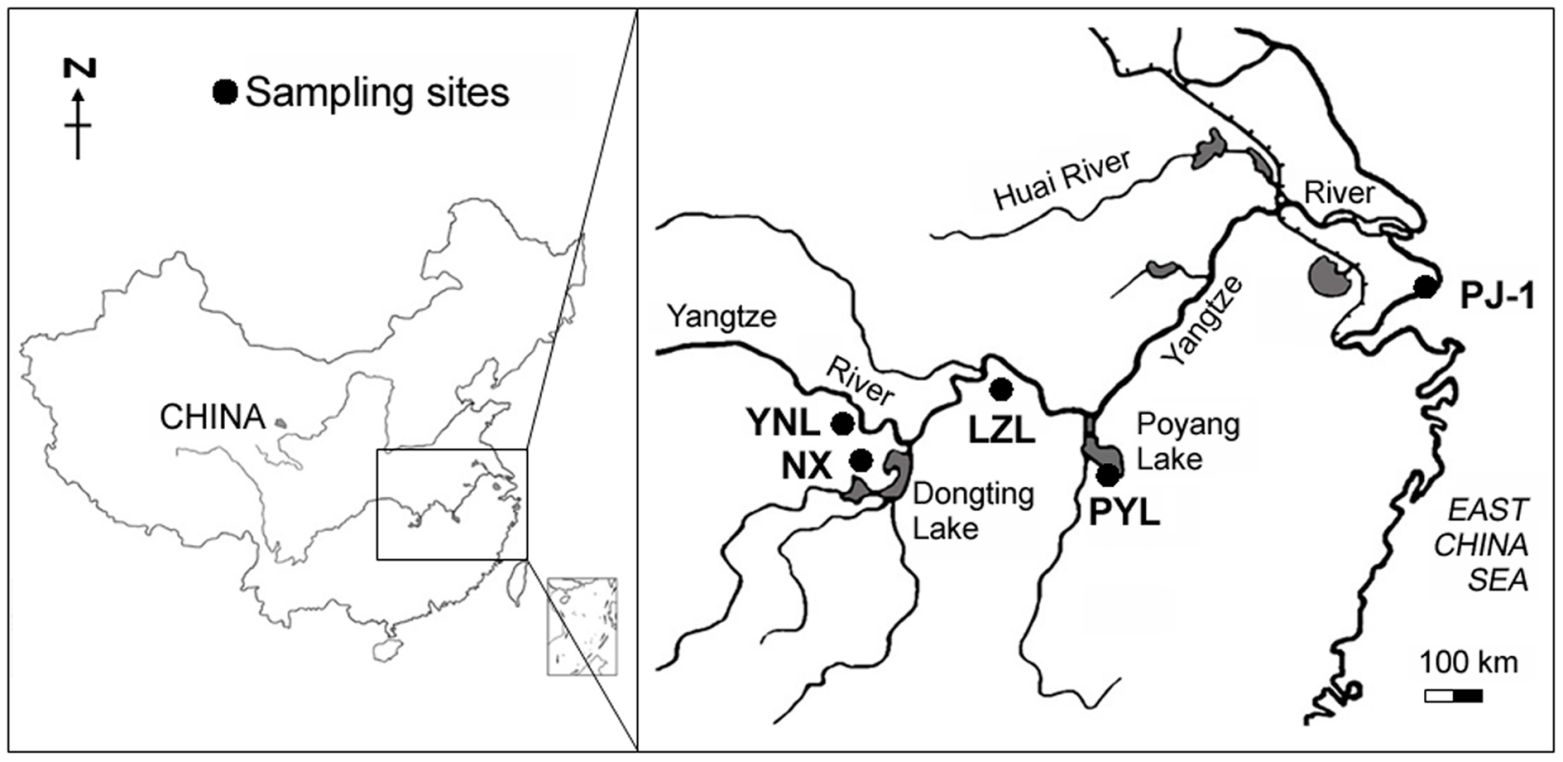
Sampling sites of *Megalobrama amblycephala* in China. Sample codes are given in Table 1.

In total, 234 individuals from five blunt snout bream populations were included in this study. Samples were collected from three natural populations (Liangzi Lake, Poyang Lake and Yuni Lake), from one selected strain (‘Pujiang No. 1’ from Shanghai), and from one cultured population (Nanxian County in Hunan Province). Details of the sampled populations are provided in Table 1 (we use the term ‘population’ for ease of use, although we recognise that each group tested here may not be a biologically defined population) and the sampling sites are showed in Figure 1. As far as can be ascertained, the Liangzi Lake, Poyang Lake and Yuni Lake populations represent a good geographic spread of natural populations from the species' original range of distribution and have been least impacted by the translocation of fish (between these sites, from other regions, from selected strains, etc). Because of the long history of human-mediated movement of fish the identification of additional natural populations is now increasing difficult, so we have focussed on what we believe to be the best source of natural (wild) fish. For sampling, all individuals were captured from lakes or ponds with a haul seine and a piece of pectoral fin (∼0.5 cm^2^) was clipped from each individual without anesthesia. After sampling, the individuals of the selected and cultured populations were returned to their corresponding ponds, whereas the individuals of the three natural populations were transported to the Ezhou fish breeding base of Huazhong Agricultural University (HZAU) for further breeding programme. The clipped fin tissues were preserved in 95% ethanol. Total DNA was extracted from fin tissues using the phenol-chloroform method [40]. DNA concentration and quality were assessed by electrophoresis on a 0.8% agarose gel. The animal research oversight committee (AROC) of HZAU had knowledge of the fish sampling plans prior to their approval of the present animal research protocol. This study was approved by the Institutional Animal Care and Use Committees (IACUC) of HZAU.

**Table 1:** Sampling locations and sample sizes of natural, cultured and genetically selected populations of *Megalobrama amblycephala*.

Population	Code	Population type	Sampling location	Coordinates (lat., long.)	Sampling date	Sample size
Liangzi Lake	LZL	natural	Liangzi Lake, Hubei Province	30°16′N, 114°31′E	Jan 2009	47
Poyang Lake	PYL	natural	Poyang Lake, Jiangxi Province	28°36′N, 116°11′E	Jan 2009	48
Yuni Lake	YNL	natural	Yuni Lake, Hubei Province	29°50′N, 112°08′E	Jan 2009	48
‘Pujiang No. 1’	PJ-1	genetically selected strain (F_7_)	Songjiang fish breeding station, Shanghai	31°02′N, 121°05′E	July 2010	48
Nanxian	NX	cultured	Shuanghu fish farm, Nanxian county, Hunan Province	29°20′N, 112°18′E	July 2010	43

### SRAP reactions

A set of 19 SRAP primers (eight forward and eleven reverse, see Table 2) was obtained from Li and Quiros [27], giving a total of 88 primer pair combinations to be used to search for polymorphisms among all individuals. Thirteen polymorphic primer pairs were used for PCR amplification of fish from all five populations because, after preliminary testing, only these primers provided informative and repeatable results.

**Table 2:** Primer sequences of SRAP markers used in this study.

Forward Primer	Sequence (5′-3′)	Reverse Primer	Sequence (5′-3′)
Me1	TGAGTCCAAACCGGATA	Em1	GACTGCGTACGAATTAAT
Me2	TGAGTCCAAACCGGAGC	Em2	GACTGCGTACGAATTTGC
Me3	TGAGTCCAAACCGGAAT	Em3	GACTGCGTACGAATTGAC
Me4	TGAGTCCAAACCGGACC	Em4	GACTGCGTACGAATTTGA
Me5	TGAGTCCAAACCGGAAG	Em5	GACTGCGTACGAATTAAC
Me6	TGAGTCCAAACCGGTAA	Em6	GACTGCGTACGAATTGCA
Me7	TGAGTCCAAACCGGTCC	Em7	GACTGCGTACGAATTCAA
Me8	TGAGTCCAAACCGGTGC	Em8	GACTGCGTACGAATTCTC
		Em9	GACTGCGTACGAATTCGA
		Em10	GACTGCGTACGAATTCAG
		Em11	GACTGCGTACGAATTCCA

All SRAP PCR reactions were carried out in a final volume of 15 µL containing 1.5 µL 10×PCR buffer, 2.0 mM Mg^2+^, 0.25 mM dNTP, 0.6 U *Taq* DNA polymerase (Fermentas), 0.5 µM each of primers, and 20 ng template DNA. All PCRs were conducted in a PTC-200 Thermocycler under the following conditions: an initial denaturing step was performed at 94°C for 5 min, followed by five cycles of 1 min at 94°C, 1 min at 35°C, 1 min at 72°C, then 35 cycles at 94°C for 1 min, 50°C for 1 min, 72°C for 1 min, followed by a final extension step at 72°C for 10 min. PCR products were separated on a 6% non-denaturing polyacrylamide gel via electrophoresis and silver staining was used to visualize the results.

### Data analysis

Using a 100 bp DNA marker ladder as a size standard, SRAP size-dependent fragments were scored as absent (0) or present (1) to obtain a binary matrix. Only clear, strong and reproducible bands in the size range of 60 to 1124 bp were scored. POPGENE 1.32 was used to calculate number of polymorphic bands (*N*), percent of polymorphic bands (*P*), Nei's gene diversity (*h*), Shannon's information index (*I*), Nei's unbiased genetic distance (*D*) and genetic similarity (*S*) estimates [41]. Based on Nei's *D*, a NJ tree with bootstrap support values (10,000 replications) was constructed using POPTREE2 [42] to illustrate the relationship among populations. Genetic population structure was quantified by analysis of molecular variance (AMOVA) using ARLEQUIN 3.11 [43]. The pairwise fixation index (*F*_ST_) was used to evaluate the level of genetic differentiation among populations. Sequential Bonferroni corrections [44] were used to correct for multiple testing.

To further evaluate the extent of genetic differentiation among the five populations we carried out a discriminant function analysis (DFA) to determine the population assignment success for each individual fish based on its genetic diversity. The SRAP markers had 140 variable bands, a number too large for the DFA to work with, so we summarised the genetic variation using principal components analysis (PCA). The first 40 eigen vectors, which explained 82% of the variation in the SRAP data set, were used in the DFA. The DFA was based on original group size (i.e., the number of fish per population that was actually sampled). The DFA and PCA were carried out using STATISTICA v7 software (StatSoft Ltd).

## Results

### SRAP analysis

Eighty-eight primer combinations were tested and of these, 13 primer pairs (Me1/Em3, Me1/Em4, Me1/Em6, Me1/Em7, Me4/Em1, Me4/Em2, Me4/Em8, Me5/Em1, Me5/Em2, Me5/Em4, Me5/Em7, Me8/Em8, and Me8/Em9) showed clear, reproducible and polymorphic banding patterns. The number of amplified bands for each primer pair ranged from 8 to 21 (average 13). In total, 172 bands were detected, of which 132 bands showed polymorphism, giving a polymorphic value of 76.7%.

### Genetic diversity

For fish from the five populations (mean ± SD sample size of 46.8±2.17), the number of polymorphic bands (*N*) and the percentage of polymorphic bands (*P*) were highest in the PYL population (*N* = 100, *P* = 58.14%), and lowest in the YNL population (*N* = 50, *P* = 29.07%). Nei's gene diversity (*h*) across the five populations ranged from 0.090 (YNL) to 0.185 (PYL), and Shannon's information index (*I*) ranged from 0.137 (YNL) to 0.280 (PYL) (Table 3).

**Table 3:** Genetic diversity results for five populations of *Megalobrama amblycephala* based on SRAP markers.

	Population				
Parameter	LZL	PYL	YNL	PJ-1	NX
Number of polymorphic bands (*N*)	88	100	50	56	64
Percent of polymorphic bands (*P*, %)	51.16	58.14	29.07	32.56	37.21
Nei's gene diversity (*h*) (mean ± SD)	0.152±0.183	0.185±0.195	0.090±0.166	0.107±0.176	0.126±0.183
Shannon's information index (*I*) (mean ± SD)	0.235±0.265	0.280±0.280	0.137±0.240	0.162±0.256	0.191±0.266

### Genetic differentiation among populations

The SRAP markers revealed a high level of genetic differentiation among the five blunt snout bream populations. Pairwise *F*_ST_ values ranged from 0.351 between the geographically adjacent natural populations of Liangzi Lake (LZL) and Poyang Lake (PYL), to 0.685 between the Yuni Lake (YNL) population and the genetically selected strain of ‘Pujiang No. 1’ (PJ-1). The differences in genotype frequencies between all pairs of populations were highly significant (*P*<0.001) (Table 4).

**Table 4:** Pairwise fixation index values (*F*_ST_) between pairs of blunt snout bream (*Megalobrama amblycephala*) populations.

Population	LZL	PYL	YNL	PJ-1	NX
LZL	——				
PYL	0.351***	——			
YNL	0.533***	0.573***	——		
PJ-1	0.573***	0.572***	0.685***	——	
NX	0.544***	0.526***	0.659***	0.434***	——

Note:

***Significant at *α* = 0.001 after sequential Bonferroni correction for multiple testing.

Analysis of molecular variance (AMOVA) of hierarchical gene diversity indicated that 44.51% of the genetic variation was explained by within-population variation, whereas 55.49% of the variation was explained by among-population variation, with a significant fixation index (*F*_ST_ = 0.555, *P*<0.001) (Table 5).

**Table 5:** AMOVA analysis of genetic variation in five populations of blunt snout bream based on SRAP markers.

Source of variation	df	Sum of squares	Variance components	Percentage of variation (%)	*F*_ST_-value
Among-populations	4	2376.04	12.48	55.49	0.555***
Within-populations	229	2293.57	10.02	44.51	
Total	233	4669.61	22.50		

Note:

***Significant at *α* = 0.001 level.

Nei's unbiased genetic distances (*D*) among all populations ranged from 0.087 (LZL-PYL) to 0.270 (YNL-NX), and Nei's unbiased genetic similarities (*S*) ranged from 0.763 (YNL-NX) to 0.917 (LZL-PYL) (Table 6).

**Table 6:** Nei's unbiased genetic distance (below diagonal) and genetic similarity (above diagonal) among five populations of blunt snout bream.

Population	LZL	PYL	YNL	PJ-1	NX
LZL	——	0.917	0.831	0.837	0.844
PYL	0.087	——	0.802	0.805	0.815
YNL	0.186	0.221	——	0.776	0.763
PJ-1	0.178	0.217	0.254	——	0.916
NX	0.169	0.204	0.270	0.088	——

**Figure 2:**
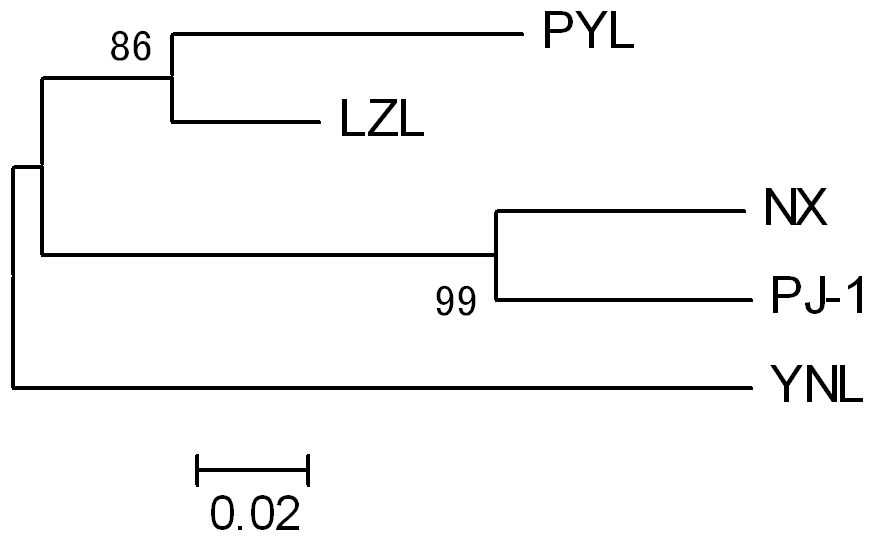
NJ dendrogram of relationships among blunt snout bream populations based on Nei's unbiased genetic distance. Percent bootstrap support values for 10,000 replications are indicated at nodes. See Table 1 for population abbreviation definitions.

The NJ dendrogram constructed using Nei's unbiased genetic distance (Figure 2) showed that the populations were grouped into three clusters. The LZL (Liangzi Lake) population clustered with its geographically close population of Poyang Lake (PYL). The genetically selected population of ‘Pujiang No. 1’ (PJ-1) and the cultured population of Nanxian (NX) clustered together but separated from LZL and PYL samples. The YNL (Yuni Lake) population formed a distinct branch, separated from all other populations. Bootstrap support values for these clusters were all very high.

Discriminant function analysis (DFA) of the first 40 eigen values from the PCA resulted in 100% correct assignment. That is, all fishes from all populations were correctly assigned to their populations of sampling. The DFA result is consistent with the *F*_ST_, AMOVA and NJ analysis, all of which reveal a high level of genetic differentiation among fish from the five populations.

## Discussion

China's freshwater fish diversity is high at 920 species in 302 genera [12]. Among the recorded species, most (73%) are Cypriniformes, and across the country's 9 freshwater regions, river discharge contributes most to explaining variation in species richness [12]. Within the Yangtze River basin itself, 361 species and subspecies have been recorded, of which 177 are endemic [2]. Twenty-five fish species have been identified as being endangered [45]. It has been suggested that hydrological alterations pose the greatest threat to the basin's fish diversity and that the most immediate restoration need is for reconnection between the Yangtze River and its lakes [2]. In concluding, Fu *et al.* noted that the cluster of lakes within the middle Yangtze River region requires protection for the preservation of migratory fishes and that there is a need to identify areas of high fish biodiversity to mitigate loss of fish biodiversity in the Yangtze River basin [2]. In our study, we focus on the genetic diversity of the blunt snout bream, a cyprinid, in the cluster of lakes in the middle Yangtze River region. The focus of our research is primarily around conservation, but we recognize that there may be tension between conservation planning and fishing/aquaculture activity, and as such our ultimate aim is to contribute new knowledge to both conservation and fisheries management.

### Genetic diversity

All four indices (number of polymorphic bands *N*, percent of polymorphic bands *P*, Nei's gene diversity *h*, Shannon's information index *I*) showed the same order from largest to smallest for the five populations. Both *h* and *I* indicated that the highest genetic diversity level was observed in the PYL population, followed by the LZL population, and the lowest was in the YNL population, all three being natural populations of *Megalobrama amblycephala*. As suggested by Li *et al.* [13], the genetic diversity level of fish within Niushan Lake (a subsidiary lake of Liangzi Lake) is a little higher than that within Yuni Lake. Our SRAP-based results are however, not in agreement with previous estimates of genetic diversity based on RAPD analysis that had shown that the genetic similarity of blunt snout bream from Yuni Lake (0.962) is very similar to that of fish from Liangzi Lake (0.959) [18]. However, mtDNA control region sequence analysis by Li [21] revealed a low level of population diversity within Yuni Lake compared to Liangzi Lake and Poyang Lake, with the haplotype diversity and nucleotide diversity index values detected in fish from Yuni Lake being only half of those for fish from Liangzi and Poyang Lakes. These mtDNA-based findings are similar to the results reported by us based on SRAP markers which represent the most up to date estimates of genetic diversity. The highest SRAP-based variation was observed for fish from PYL, which is the lake least affected by human activity (e.g., transfer of fish). The values reported for fish from this lake therefore give some indication of the natural level of genetic variability that may be expected to occur in wild fish, and also a measure of the impact (reduced genetic variability) associated with human-mediated activities such as breeding programmes, inbreeding, and transfer of fish from one lake to another.

For the cultured (Nanxian) and selectively bred (‘Pujiang No. 1’) populations, the levels of genetic diversity were much lower than within the native PYL and LZL populations. These results are in agreement with investigations on largemouth bass (*Micropterus salmoides*) and large yellow croaker (*Pseudosciaena crocea*) [46], [47], and are consistent with the known impacts of hatchery-based breeding programmes in general ([9], [10] and references therein). In summary, as a result of artificial selection, the chance of the effective number of individuals contributing to the population declines, with an associated decline in population genetic diversity and an increase in inbreeding depression.

An unexpected outcome of our research is that the genetic diversity level of fish from the cultivated and breeding populations was found to be greater than that of wild blunt snout bream from one native lake population (Yuni Lake, YNL). This surprising result may be due to the degradation of germplasm resources of YNL over a reasonably short period of time. Previously, Li *et al.* [13] and Zhang [18] reported that the genetic diversity of blunt snout bream from Yuni Lake was similar to that of fish from Liangzi Lake at the beginning of the early 21st century. Subsequently, Li [21] reported that the genetic diversity of fish from Yuni Lake was nearly 50% of the level of fish from Liangzi Lake, a sharp decline of genetic diversity of fish from Yuni Lake. Whilst different marker types (e.g., allozymes, mtDNA sequencing) have been used by the different studies, each study is internally consistent and points to a decrease in genetic diversity of fish from Yuni Lake over a short period of time. Our study has revealed that the genetic diversity of blunt snout bream from YNL is lower than that of a cultured population and a selectively bred population. We suggest that the results obtained for fish from YNL are attributable to the rapid intensification of artificial culture, propagation, and over-fishing of blunt snout bream in Yuni Lake during the past 10 years, which have resulted in much reduced genetic diversity of bream germplasm at this location. Whilst further research is needed to determine if this is actually the case, our research highlights the potential negative impact of culture, selective breeding programmes and multiple transfer of fishes on native genetic diversity of this species of fish in lakes of the middle Yangtze River region.

### Population differentiation

*F*_ST_ is one of the most widely used measures for evaluating the genetic differentiation between/among populations, which can provide important insights into the evolutionary processes that cause the genetic differentiation among populations [48]. Whilst the magnitude of *F*_ST_ values is to some extent species- or even group-specific, as a general rule *F*_ST_ values of 0–0.05 represent little differentiation, values of 0.05–0.25 indicate moderate differentiation, and values higher than 0.25 indicate very great differentiation among populations [49]. In the present study, all values of *F*_ST_ between the five populations were >0.25, indicative of a high level of genetic differentiation among these five populations. The highest *F*_ST_ value detected (0.685) was between PJ-1 and YNL, suggesting that pronounced genetic differentiation now exists between the selectively bred strain (PJ-1) and the natural YNL population. These findings were supported by our discriminant function analysis (DFA) which was able to assign each and every fish to its population of origin, are comparable to the results reported by [50], and indicate how rapidly genetic differentiation can be achieved between wild breeding and selectively bred fishes (the PJ-1 fish are from an F_7_ strain). The five populations of blunt snout bream clustered into three groups, as revealed by NJ analysis. Two of the three wild populations, PYL and the geographically close population of LZL, clustered together, while the third wild population (YNL) was highly differentiated from the other two wild populations (in the NJ analysis YNL is identified as a distinct branch). In addition, we tried to construct a NJ tree based on pairwise *F*_ST_ between populations at the same time. The NJ tree based on pairwise *F*_ST_ was consistent with the results above (data not shown). These results confirm earlier observations that obvious genetic divergence occurred between fish from YNL and LZL or PYL, and no divergence occurred between LZL and PYL [21]. Historically, PYL, LZL and YNL (that is, Poyang Lake, Liangzi Lake, and Yuni Lake, respectively) were linked to the Yangtze River. Yuni Lake was separated from the Yangtze River about forty years ago due to human activities [13], which isolated its fish and has prevented genetic exchange among populations that were previously connected. This isolation has resulted in a build-up of genetic differentiation between fish from Yuni Lake and the two other lakes (i.e., there has been a brief but substantial period of divergence between the fish populations). LZL and PYL were still connected via the Yangtze River and closer to each other geographically, so there was still gene flow between these two lakes, therefore a closer genetic relationship was found between the populations from these two lakes. Whilst fish from these two lakes exhibit the highest levels of pairwise similarities, they are still sufficiently genetically differentiated to be distinct. These findings support the contention of Fu *et al.* [2] that lakes within the central Yangtze River region are in need of further protection and that connections between them and the river itself should be re-established. Finally, the genetically selected population of PJ-1 and the cultured population of NX clustered together to form a third, separate group that lies between the two previously described wild lake groups. The PJ-1 fish were produced through long-term artificial selective breeding since 1986, based on original wild population of blunt snout bream from Yuni Lake [26]. This selectively-bred line now has a growth rate that is increased by 30% compared to wild fish [17], [51]. The distinct genetic differences among the three groups as revealed by NJ analysis indicate that genetic divergence has occurred between the selective breeding population (PJ-1), the cultured population (NX) and the wild populations (LZL, PYL and YNL). Similar results have also been reported for grass carp and mandarin fish [35], [52]. This finding illustrates that restocking of lakes by fish from selectively bred programmes should be carried out with great care and a complete understanding of the genetic diversity that may exist between fish strain and recipient lake, if extensive interbreeding between donor strain and wild lake fish is expected.

In conclusion, the SRAP system has been successfully employed to assess population genetic diversity among domesticated and wild genotypes of blunt snout bream from China. Analysis revealed that cultivated, genetically selected and wild bream germplasm are highly differentiated and that wild lake populations have high levels of genetic diversity. Comparison of the genetic diversity of wild fish to that of cultured and selectively bred fish illustrates the need for greater care managing this important resource so that the aquaculture programme to support this species is not disadvantaged in the future and so that wild populations of fish are protected for both conservation and fisheries purposes.
